# A University Hospital Based Study on Thoracic Trauma: Life Threatening Event, Its Etiology, Presentation, and Management

**DOI:** 10.7759/cureus.6306

**Published:** 2019-12-06

**Authors:** Raheel Ahmad, Dujanah S Bhatti, Muhammad Haseeb T Bokhari, Ayesha Asad

**Affiliations:** 1 Surgery, Holy Family Hospital, Rawalpindi, PAK; 2 Plastic Surgery, Holy Family Hospital, Rawalpindi Medical College, Rawalpindi, PAK; 3 Surgery, Holy Family Hospital, Rawalpindi Medical College, Rawalpindi, PAK; 4 Anatomy, Quetta Institute of Medical Sciences, Quetta, PAK

**Keywords:** thoracic trauma, road traffic accidents, pneumothorax, tube thoracostomy, empyema thoracis

## Abstract

Introduction: Thoracic injury is becoming an important cause of mortality in Pakistan, especially in the younger subset of population. The initial management of these injuries is essential as these patients can develop multiple complications, ultimately leading to death of the patients.

Materials and methods: This prospective observational study was carried out from January 2016 to December 2018 at the Department of Surgery, Holy Family Hospital, Rawalpindi Medical University, Pakistan. It included all the patients over 12 years of age who had thoracic trauma, who presented in the ED, and were admitted to the surgical ward and intensive care unit (ICU). Data were collected with the help of a pre-designed proforma. After relevant investigations and treatment, data were collected and analyzed through Statistical Package for Social Sciences (SPSS) for version 19. Nominal variables were reported as frequencies and percentages.

Results: Out of a total of 330 patients, 188 (56.9%) suffered from blunt injuries whereas 142 (43%) had penetrating injuries. The most common cause of these injuries was road traffic accidents -- 105 (32%) followed by falls -- 23 (76%). Most of the injuries encountered were isolated pneumothorax -- 74 (22.4%) followed by rib fractures with pneumothorax -- 71 (21.5%). Tube thoracostomy was done in 189 cases (57.3%) whereas 94 (28.5%) patients were managed conservatively. Complications were seen in 117 patients (35.4%). Out of these 117 cases, death was the major complication - 30 (25.6%) followed by bronchopleural fistula - 24 (20.5%) and empyema thoracis - 22 (18.8%).

Conclusion: Road traffic accidents are a major cause of thoracic injuries in our setting. Tube thoracostomy is the most commonly used treatment modality. Mortality rate is high in the patients with thoracic injuries.

## Introduction

Trauma is one of the leading causes of morbidity and mortality throughout the world. In the United States, about 79,000 deaths are attributed to trauma in the younger population [1]. Thoracic trauma is emerging as one of the major findings in the trauma settings and comprises 10%-15% of all trauma cases. It can be divided into different types depending upon the location of the injury. The major locations include injury to the chest wall, lungs, esophagus, heart, and major vessels. The initial management of these injuries is essential as these patients can develop multiple complications, ultimately leading to death of the patients. This is evident by the fact that about 25% of the trauma-related deaths are due to thoracic trauma [2]. 

In Pakistan, thoracic injury is also becoming an important cause of mortality especially in the younger subset of population. The incidence of blunt injuries is higher than the penetrating thoracic injuries. There are only a few studies available demonstrating the different aspects of thoracic trauma in this region of the world [3-4].

The objective of our study was to do a thorough analysis of thoracic trauma in a tertiary care hospital in Pakistan. The points that are highlighted in this study are etiology, types, complications during the hospital stay, and management of the thoracic trauma.

## Materials and methods

This was a prospective observational study carried out during January 2016 to December 2018 at the Department of Surgery, Holy Family Hospital, Rawalpindi Medical University, Pakistan. It included all the patients over 12 years of age who had thoracic trauma, who presented in the ED, and were admitted to the surgical ward and intensive care unit (ICU).

Data were collected with the help of a pre-designed proforma that included patients’ demographic profile, age, gender, cause of trauma, presenting symptoms, various chest and other associated injuries, underlying co-morbidities, interventional procedures undertaken, complications, and mortality. The relevant investigations which were carried out at presentation and during hospital stay were chest X-ray, focused assessment with sonography for trauma (FAST) scan, electrocardiography (ECG), cardiac enzymes, arterial blood gases (ABGs), CT scan chest, bronchoscopy, endoscopy, and echocardiography.

Tube thoracostomy was done for all the patients with pneumothorax, hemothorax, hemopneumothorax, surgical emphysema, pneumomediastinum, and tracheobronchial injuries.

All penetrating cardiac injuries underwent thoracotomy while the rest of the thoracotomies were done for other indications either immediately in emergency operation theater or in the elective operation theater after an interval. Video-assisted thoracoscopic surgery (VATS) was done for clotted hemothorax or empyema thoracis. All the rib fractures were managed conservatively without any surgical intervention. Acute respiratory distress syndrome (ARDS) was labeled when the ratio of partial pressure of arterial oxygen to fraction of inspired oxygen (PaO2/FiO2) was 200 or less, regardless of positive end-expiratory pressure, bilateral infiltrates seen on frontal chest radiograph and pulmonary artery wedge pressure of 18 mmHg or less with no clinical evidence of left atrial hypertension.

The data were analyzed through Statistical Package for Social Sciences (SPSS) for version 19. Nominal variables were reported as frequencies and percentages.. 

## Results

**Figure 1:**
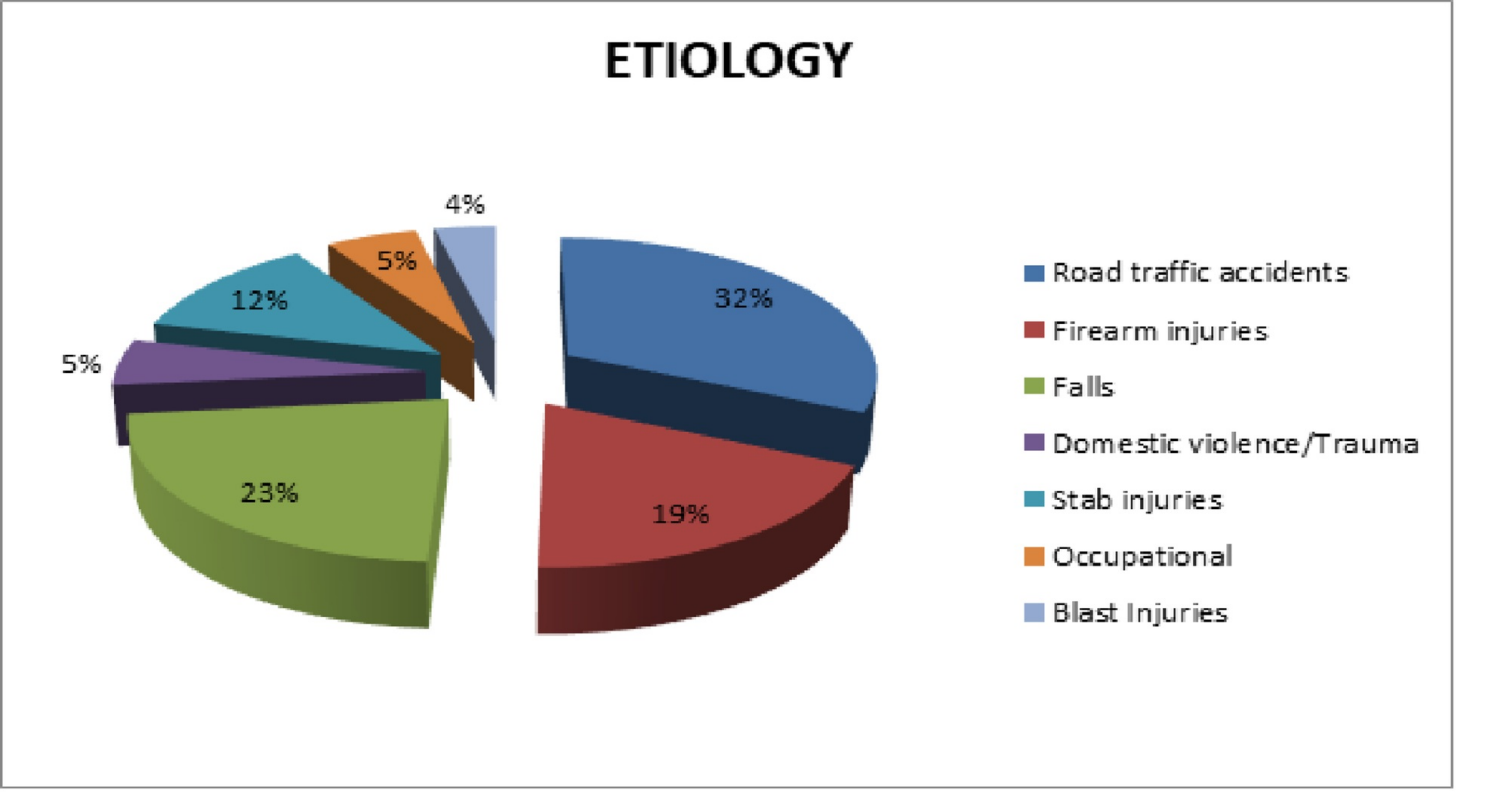
Etiology of thoracic trauma (n = 330).

A total of 330 patients were admitted in our ward with thoracic injuries. One hundred and eighty-eight (56.9%) of the injuries were blunt injuries whereas 142 (43%) were penetrating injuries. The most common cause of these injuries was road traffic accidents -- 105 (32%) followed by falls -- 23 (76%). Thirteen (4%) cases were attributed to blast injuries as shown in Figure 1.

Most of the injuries encountered were isolated pneumothorax -- 74 (22.4%) followed by the rib fractures with pneumothorax -- 71 (21.5%). Hemothorax constituted 58 (17.6%) of the cases. Twenty-five (7.6%) of the cases had isolated rib fractures. Clavicular and scapular injuries were 19 (5.7%) and 17 (5.1%) of the total cases, respectively. There was also one case of esophageal injury. Other types of thoracic injuries along with their distribution as blunt and penetrating injuries are shown in Table 1.

**Table 1:** Types of thoracic injuries (n = 330).

Type of injury	Blunt (n = 188)	Penetrating (n = 142)	Total (%)
Rib fracture with pneumothorax	46	25	71 (21.5%)
Isolated rib fracture	25	00	25 (7.6%)
Isolated pneumothorax	28	46	74 (22.4%)
Hemothorax	23	35	58 (17.6%)
Clavicular fracture	17	02	19 (5.7%)
Scapular fracture	11	06	17 (5.1%)
Multiple fractures	09	05	14 (4.2%)
Flial chest	03	04	07 (2.1%)
Pulmonary contusion	15	00	15 (4.5%)
Major vascular & cardiac injuries	01	04	05 (1.5%)
Tracheo bronchial injuries	07	07	14 (4.2%)
Diaphragmatic injuries	03	07	10 (3.03%)
Esophageal injury	00	01	01 (0.3%)

Out of 330 patients, 189 (57.3%) were managed by tube thoracostomy whereas 94 (28.5%) of the patients were managed conservatively. Nineteen cases (5.7%) cases required ventilator support while 17 (5.1%) of the cases needed thoracotomy. Operative fracture management was done in two (0.6%) of the patients. Management of the thoracic injuries according to blunt and penetrating injuries is shown in Table 2.

**Table 2:** Management of thoracic injuries (n = 330). VATS, video-assisted thoracoscopic surgery

Management	Blunt (n = 188)	Penetrating (n = 142)	Total (%)
Tube thoracostomy	91	98	189 (57.3%)
Conservative management	71	23	94 (28.5%)
Ventilatory support	14	05	19 (5.7%)
VATS	05	04	09 (2.7%)
Thoracotomy	05	12	17 (5.1%)
Operative fracture management	02	00	02 (0.6%)

Complications were encountered in 117/330 (35.4%) cases. Out of these 117 cases, 61 (52.1%) had blunt injuries while 56 (47.9%) had penetrating injuries. Thirty patients (25.6%) died after thoracic injury. Twenty-four patients (20.5%) developed bronchopleural fistula while 22 (18.8%) developed empyema thoracis. ARDS was found in 21 (17.9%) patients. Respiratory failure and clotted hemothorax was found in 11/117 (9.4%) and 9/117 (7.7%) patients respectively. Complications of blunt and penetrating thoracic injuries are shown in Table 3.

**Table 3:** Complications of thoracic trauma during hospital stay (n = 117). ARDS, acute respiratory distress syndrome

Complication	Blunt (n = 61)	Penetrating (n = 56)	Total (%)
Empyema thoracis	09	13	22 (18.8%)
Bronchopleural fistula	09	15	24 (20.5%)
ARDS	15	06	21 (17.9%)
Clotted hemothorax	06	03	09 (7.7%)
Respiratory failures	08	03	11 (9.4%)
Death	14	16	30 (25.6%)

## Discussion

Chest injury is a common cause of morbidity, prolonged hospital stay, and mortality in the people of productive age group. The etiology of chest trauma varies slightly according to the study population and location. The major cause of chest trauma in this study was road traffic accidents responsible for 32% of the trauma victims. This was consistent with the findings of several studies including Lema et al. and Ustundag et al. [5-6]. Falls and gunshot wounds were the next most common causes responsible for 23% and 19% of trauma patients respectively. Falls were also found to be the second most common cause of chest trauma by Veysi et al. [7].

Road traffic accidents causing a high proportion of chest injuries point towards the worrying side effects of urbanization. Disregard of traffic rules, particularly in this part of world results in the exacerbation of the problem and effective legislation is required to help improve the situation.

The type of injury is important in formulating the treatment plan. The most common injury reported in our study was isolated pneumothorax (22.4%) followed by pneumothorax with rib fractures (21.5%). Isolated pneumothorax was a much common finding in the penetrating wounds while rib fractures were relatively more common in the blunt injuries. Similar findings were reported by Veysi et al. and Elbaih [7-8]. A study in Nigeria showed rib fractures as most common injury followed by hemopneumothorax [9].

Early and effective treatment of chest injuries is vital for improving the long-term survival of trauma victims. Nonoperative management is the most advocated and widely used treatment option used worldwide. Majority of patients in our setting were treated with tube thoracostomy (57.3%) with the next most common treatment option being conservative management (28.5%). Multiple studies from all over the globe reported conservative management and thoracostomy as the primary treatment modalities [10-13].

 Thoracostomy is a lifesaving procedure as it provides maximum benefit in a short time with minimal complications. This procedure, due to its relative simplicity and effectiveness, has become the treatment of choice in the trauma patients requiring chest drainage. 

Appearance of complications is an effective indicator of prognosis and may influence the long-term survival of the trauma patients. Complications were encountered in 117 (35.4%) cases. The two major complications were bronchopleural fistula (20.5%) and empyema thoracis (18.8%). Lema et al. reported wound sepsis as the number one complication, while empyema thoracis was comparatively seen in very fewer cases [5].

Mortality rate in this study was high. Overall 30 deaths occurred, making up 9.1% of the total cases. Lema et al. have reported a lower mortality rate (4.7%) [5]. The mortality rate was reported to be higher by Atri et al. and Massaga et al. [14-15]. Overall mortality rate is influenced by early management and availability of specialized equipment to handle the complications. Effective first aid measures provide valuable assistance to the health team and may help improve the overall prognosis of the trauma victims.

## Conclusions

Thoracic trauma is quite prevalent in trauma settings. In our study, the prevalence of blunt thoracic injuries was higher than the penetrating injuries. The most common etiology was road traffic accidents, with falls and blast injuries being second and third in the list, respectively. The most common types of injuries associated with this trauma were isolated pneumothorax followed by rib fracture with pneumothorax. In the management, the most common procedure performed was tube thoracostomy with conservative management second in the list. The common complications included death, bronchopleural fistula, and empyema thoracis. Further studies are required for a detailed understanding of the thoracic trauma and its implications.
